# Optimization of the magnetic labeling of human neural stem cells and MRI visualization in the hemiparkinsonian rat brain

**DOI:** 10.1186/s12951-015-0078-4

**Published:** 2015-03-05

**Authors:** Milagros Ramos-Gómez, Emma G Seiz, Alberto Martínez-Serrano

**Affiliations:** Centre for Biomedical Technology, Polytechnic University of Madrid, 28223 Madrid, Spain; Biomedical Research Networking Center in Bioengineering Biomaterials and Nanomedicine (CIBER-BBN), Madrid, Spain; Department of Molecular Biology and Center of Molecular Biology “Severo Ochoa”, Autonomous University of Madrid-C.S.I.C, 28049 Madrid, Spain

**Keywords:** Magnetic nanoparticles, Neural stem cell, Cell tracking, Magnetic resonance imaging

## Abstract

**Background:**

Magnetic resonance imaging is the ideal modality for non-invasive *in vivo* cell tracking allowing for longitudinal studies over time. Cells labeled with superparamagnetic iron oxide nanoparticles have been shown to induce sufficient contrast for *in vivo* magnetic resonance imaging enabling the in vivo analysis of the final location of the transplanted cells. For magnetic nanoparticles to be useful, a high internalization efficiency of the particles is required without compromising cell function, as well as validation of the magnetic nanoparticles behaviour inside the cells.

**Results:**

In this work, we report the development, optimization and validation of an efficient procedure to label human neural stem cells with commercial nanoparticles in the absence of transfection agents. Magnetic nanoparticles used here do not affect cell viability, cell morphology, cell differentiation or cell cycle dynamics. Moreover, human neural stem cells progeny labeled with magnetic nanoparticles are easily and non-invasively detected long time after transplantation in a rat model of Parkinson’s disease (up to 5 months post-grafting) by magnetic resonance imaging.

**Conclusions:**

These findings support the use of commercial MNPs to track cells for short- and mid-term periods after transplantation for studies of brain cell replacement therapy. Nevertheless, long-term MR images should be interpreted with caution due to the possibility that some MNPs may be expelled from the transplanted cells and internalized by host microglial cells.

## Background

Neural stem cells (NSCs) represent a source of cells for regenerative medicine, in particular for cell replacement therapies both in the clinical and pre-clinical experimental settings. One of the goals of stem cell research is the *in vitro* and *in vivo* generation of neurons which could turn to be optimal candidates to replace specific lost neurons, for instance in Parkinson’s disease (PD), in which the A9 subtype of dopaminergic neurons (DAn) in the Substantia nigra (SN) are lost [1]. Previous clinical studies of cell replacement in PD were based on the transplantation of fresh human fetal ventral mesencephalic (VM) tissue into the caudate and putamen of PD patients [1,2]. These initial experiments showed practical and ethical issues such as the need to obtain tissue from six to seven human fetuses to provide enough cells for one patient’s transplantation, the lack of reproducibility between centers, poor survival in some cases, and the appearance of serious adverse side-effects in some patients. Recent work has thus aimed to obtain suitable sources of human NSCs (hNSCs) with the capacity to differentiate into DAn endowed with the required, genuine properties of Substantia Nigra pars compacta neurons (SNpc) lost in PD [3,4].

Recent pre-clinical research has demonstrated that immortalized human NSCs, derived from VM (hVM1 cell line) and modified for the elevated expression of Bcl-XL (hVM1-highBcl-XL cells), have the potential to differentiate into DAn *in vivo* at a high rate [5-9]. After transplantation in hemiparkinsonian rats, these hVM cells survive, integrate, and differentiate into DAn, alleviating behavioral motor asymmetry and skilled paw use [5,9,10]. Thus, hVM1 cells and their derivatives represent a helpful tool for the development of cell therapies for neurodegenerative diseases, Parkinson disease in particular.

Tracking noninvasively the long-term spatial destination and final residence of transplanted cells *in vivo*, to monitor their survival, migration, differentiation and regenerative impact, has become a critical methodology in evaluating the efficacy of stem cell therapy procedures. Until now, it was only the behavioral testing or the DA determination by *in vivo* HPLC and the subsequent histological analysis the available methods used to evaluate grafting outcome, viability and differentiation of transplanted cells in hemiparkinsonian animal models. But, optimally, research in cell replacement therapy requires of non-invasive and sensitive imaging techniques to track the fate of transplanted cells; these techniques would increase reliability and reduce the total number of animals used in these experiments.

Labeling cells with magnetic nanoparticles (MNPs) has been shown to induce sufficient contrast for magnetic resonance imaging (MRI) of cells in the brain [11-15]. Therefore, MRI, in combination with other *in vivo* molecular imaging techniques, like PET, can provide insights into different cellular processes, including localization and migration of the cells, cell survival and proliferation kinetics, and cell differentiation patterns, which can aid clinical implementation of cell therapy [16].

Most labeling techniques currently take advantage of either the attachment of MNPs to the stem cell surface or the internalization of MNPs by endocytosis. Surface labeling normally results in lower iron content per cell and promotes a rapid reticulo-endothelial recognition and clearance of labeled cells [17,18]. Therefore, endocytosis of MNPs during *in vitro* cell cultivation stands as the preferred labeling method.

The most commonly used MNPs to label cells, dextran coated superparamagnetic iron oxide (SPIO) nanoparticles, as the ones used in the present study, do not efficiently label either nonphagocytic or non–rapidly dividing mammalian cells in vitro [19]. Consequently, these contrast agents are not used as isolated reagents to label hNSCs or other mammalian cells [20-22]. In most cases, internalization of nanoparticles by hNSCs requires the use of transfection agents (TAs), like protamine sulfate (PS) or poly-L-lysine (PL) to achieve an efficient labeling of the stem cells. TAs coat MNPs by means of electrostatic interaction with dextran-coated nanoparticles and help internalizing them into cells. PS, conventionally used to reverse heparin anticoagulation, has been used as a cationic TA to label human mesenchymal stem cells and hematopoietic stem cells with SPIO nanoparticles [19]. The use of PL complexed with MNPs also reported a high labeling efficiency of NSCs, 80% [23]. However, the use of TAs to label cells might have a harmful side effect decreasing cell viability, since most TAs are toxic to cells when used alone and not complexed to DNA [19]. In addition, the use of relative high concentrations of magnetic nanoparticles to label hNSCs might be toxic or affect some of their functional properties, causing alterations in their differentiation processes. Thus, an extensive study of the properties of NSCs labeled with MNPs must be carried out to identify the effects of MNPs on hNSCs biology.

The aim of the present study was to efficiently label cells using magnetic nanoparticles detectable by MRI and to determine the effects of such particles on morphology, cell cycle and differentiation capacity of hVM cells. Here we report the development and validation of an efficient protocol to label hNSCs using several commercial magnetic nanoparticles as contrast agents for MRI. We optimized the incubation times and the concentration of MNPs to label hNSCs in the absence of any transfection agents that could damage the cellular integrity, in order to prevent impairment of the cells’ functional properties.

Our results demonstrate that the use of MNPs to label hNSCs is feasible, efficient and safe for MRI tracking following grafting of hNSCs into hemiparkinsonian rat brain. The fate of MNPs-labeled hNSCs grafted into hemiparkinsonian rats can be successfully visualized using MRI at different time points, up to 5 months after transplantation.

## Materials and methods

### Cell cultures

Cell isolation and immortalization were described previously by Villa [8] and Courtois [9]. Briefly, human ventral mesencephalic cells were isolated from a 10-week-old aborted fetus (Lund University Hospital). Tissue procurement was in accordance with the Declaration of Helsinki and in agreement with the ethical guidelines of the European Network of Transplantation. Immortalization was carried out by infection with a retroviral vector coding for v-myc (LTR-vmyc-SV40p-Neo-LTR) generating the hVM1 cell line. The hVM1 polyclonal cell line was infected at passage 6 with a Bcl-XL coding (LTR-Bcl-XL-IRES-rhGFP-LTR) retroviral vector, to enhance their neurogenic capacity [24]. After infection, the cells were selected by fluorescence-activated cell sorting (FACS) generating the hVM1 Bcl-XL cell line (referred here as hVM to abbreviate). Both cell lines were routinely cultured under standard conditions as described before [8,9]. Briefly, cells were cultured on 10 μg/ml polylysine-pretreated plasticware in epidermalgrowth factor (EGF) and basic fibroblast growth factor (FGF) (20 ng/ml each; R&D Systems) supplemented chemically defined Dulbecco’s modified Eagle’s medium/F-12 medium (Glutamax (Invitrogen), 1% Albumax (Invitrogen), 50 mM Hepes (Invitrogen), 0.6% glucose, N2 supplement (Invitrogen), 1x nonessential amino acids and penicillin/streptomycin,), referred to hereafter as “proliferation medium”. To induce cell differentiation, cells were seeded at 10^5^cells/cm^2^ in proliferation medium on poly-L-lysine-treated glass coverslips. After 24 h, proliferation medium was replaced by differentiation medium without EGF or FGF, and containing 1 mM dibutyryl-cAMP (Sigma) and 2 ng/ml human recombinant glial cell-derived neurotrophic factor (GDNF) (Preprotech)). Differentiation medium was changed every second day until the end of the experiment. Cells were proliferated and differentiated at 37°C and 95% humidity in a low oxygen atmosphere (5% O_2_, 5% CO_2_, in a dual CO_2_/O_2_ incubator (Forma)).

### Uptake of MNPs by hVM cells

#### Cell labeling with MNPs

hVM cells were seeded on glass coverslips at a density of 50,000 cells/cm^2^. After 24 h with proliferation medium, different types of SPIO nanoparticles were added while varying several parameters such as concentration, pre-coating with transfection agents and the time of incubation. For these studies the following conditions were used: i) different core diameter types (all dextran coated): 250 nm (G.Kisker), 50 nm (G.Kisker), 100 nm (Endorem) and 100 nm-Cy3conjugated (Chemicell); ii) concentration: 50, 100 or 300 μgFe/ml of culture medium; iii) incubation time: 3, 6, 12, 24 or 72 h; iv) pre-treatment of MNPs with transfection agents: untreated (control) and treated with poly-lysine (0.03 μgPL/μgFe,Sigma) or protamine sulfate (0.5 μgPS/g Fe, Sigma). For pre-treatment MNPs were maintained in proliferation medium with constant stirring for 24 h at room temperature before being added to cell cultures.

Following the desired incubation time, cells were rinsed with culture medium and PBS to remove unincorporated MNPs. Subsequently, the cells were differentiated and fixed with 4% of paraformaldehyde (Merck) in 0.1 M phosphate buffer at pH 7.4 for 15 minutes at room temperature. Fixed cells were blocked for 1 h in PBS containing 10% normal horse serum, 0.25% Triton X-100 and incubated overnight at 4°C with a monoclonal antibody to mark the dextran coating of the MNPs (except in the case of 100 nm-Cy3) using an antibody anti-dextran (1:500, Stem Cell Technologies). Afterwards, cells were rinsed and incubated with an anti-mouse Cy3-conjugated secondary antibody (1:500, Jackson Immunoresearch). Last, the cells were incubated with ToPro-3 (1:750, Invitrogen) for nucleic acid staining and phalloidin A488 to mark F-actin filaments and define cell shape. The magnitude of MNPs uptake was determined as the proportion of cells with green fluorescent cytosolic dots corresponding to MNPs compared to the total number of cells identified by the nuclear counterstaining with ToPro-3 using a confocal microscope coupled to a LSM510 Axiovert200 inverted microscope (Zeiss) .

#### Cell viability assays

Cell viability in unlabeled (control) cultures and in those labeled with MNPs was evaluated by the MTT assay at day 0 and 7 of differentiation. Cells were seeded at a density of 50,000 cells/cm^2^ in 0.5 ml culture medium. After labeling cells with MNPs for 72 h, 125 μl of 5 mg/ml MTT (Sigma M-2128) were added and the incubation was left to proceed for 60 min at 37°C. Then 1 ml of DMSO was added per well to extract the formazan and absorbance at 570 nm was determined.

#### Cell cycle analysis

To analyze the effect of MNPs on cell cycle, we performed a cell cycle analysis in unlabeled and MNP-labeled cells (100 nm, 50μgFe/ml) by propidium iodide staining and flow cytometry using the technique of Nicoletti [25]. The cells were seeded at a density of 30,000 cells/ cm^2^. After 24 and 48 h of incubation with the MNPs, the cells were trypsinized and washed in PBS (without Ca/Mg^2+^). Cells were centrifuged 10 min at 1000 rpm at 4°C and the cell pellets were mixed with 1 ml of cold 70% ethanol using the vortex. After18h fixation in ethanol at -20°C the samples were resuspended carefully and centrifuged 5 min at 1500 rpm. The supernatant was discarded and the pellet was resuspended in 0.5 ml of buffer cycle (50 μg/ml propidium iodide, 0.1% sodium citrate, 50 μg/ml ribonuclease A in PBS without Ca^2+^ or Mg^2+^), and incubated 30 min at room temperature for staining. The cells were then analyzed by flow cytometry (flow cytometer FACSCalibur, Becton Dickinson) using the 488 nm argon laser for excitation and filter 585/42 nm for the collection of emission (channel FL-2). The intensity of fluorescence represented in linear scale (cell cycle distribution) and data were analyzed for quantification of the regions sub-G0-G1 (less than 2n of DNA and fragmented DNA), G0-G1 (2n DNA) and S-G2-M (mitotic phase active, i.e. an amount of DNA between 2n and 4n). A total of 10.000 events were acquired and analyzed using FloJo7 software. Samples were run in biological triplicates.

#### Immunocytochemistry

Unlabeled cells and cells labeled with MNPs (50 and 100 nm size; 50μgFe/ml) for 72 h, were grown on glass coverslips in differentiation medium for the specified time (4 and 7 days) and fixed with paraformaldehyde (PFA, Merck) at 4% in 0.1 M phosphate buffer at pH 7.4, for 15 minutes at room temperature. After 3 washes in PBS, samples were blocked for 1 h with a solution of PBS containing 10% horse serum (HS) (Gibco/Life Tecnhologies) or goat serum (Gibco/Life Technologies) and 0.25% nonionic detergent Triton X-100 (Merck). Subsequently, cells were incubated overnight at 4°C with primary antibodies dissolved in a PBS solution with 0.25% triton and 1% horse-goat serum. To evaluate the possible effect of the MNPs in cell differentiation the following antibodies were used: nestin (day 0, 1:1000, Abcam; 1:500 BD Bioscience) and β-III-tubulin (day 7, 1:1000, Sigma), TH (day 7, 1:1000sigma) and GFAP (day 7, 1:1000 Sigma). To study the intracellular localization of MNPs the following antibodies were used: anti-manosidase II (day 7, 1:100, Millipore), anti-EEA1 (day 7, 1:200, BD Transduction) and anti-CD63 (day 7, 1:100, DSHB). After removing the solution with primary antibody and rinsing, the samples were incubated with secondary antibody conjugated with different fluorophores in PBS for 30 min at room temperature (1:500, Cy3, Cy5 or Alexa488, Jackson Inmunoresearch). Finally, nuclei were counterstained with Hoechst 33258 (0.2 μg/ml in PBS, Molecular Probes) or To-Pro-3 (1:500, Invitrogen). After the labeling, the coverslips were washed in PBS and distilled H_2_O, allowed to dry and mounted with Mowiol (Calbiochem).

#### MNP labeling decay with passages in culture

Cells were labeled with MNPs (50 and 100 nm-Cy3 50μgFe/ml) for 72 hours in p60 plates (3x10^4^ cells/cm^2^). Subsequently, the cultures were trypsinized and seeded on glass coverslips (passage 0) and split to new p60 plates. After two days, cells of these plates were trypsinized and seeded on glass coverslips (passage 1), remaining cells were split to new p60 plates after 48 h in culture and seeded at the same initial density (3x10^4^ cells/cm^2^). We proceeded up to the passage 4. Cells were fixed and immuno-stained to detect dextran, Phalloidin A488 and To-Pro-3. Then, the percentage of cells containing MNPs at each passage was determined using a LSM510 laser confocal Microscope coupled to an Axiovert200 (Zeiss) inverted microscope.

### Animal Experimentation

#### Lesion and Transplantation Procedures

Experiments were carried out according to the guidelines of the European Community (Directive 86/609/ECC, Directive 2010/63/EU) observing the 3Rs principles, and in accordance with the Society for Neuroscience recommendations. Animals used in this study were 3-month-old female Sprague-Dawley rats (Harlan), weighing 200–250 g at the beginning of the experiment, housed in a temperature- and humidity-controlled room, under 12-h light/dark cycles, with *ad libitum* access to food and water. Cells (in proliferative state) for transplantation in intact brains were dispersed and resuspended in Hanks’ balanced salt solution (Invitrogen) at a density of 10^5^ cells/μl. Cell suspensions (3x10^5^ cells in 3 μl) were injected into the left striatum (control unlabeled cells) and in the right striatum (cells labeled with 100 nm MNPs-Cy3 at 50 μg/ml for 72 h) at the following coordinates (in mm): anteroposterior -1; mediolateral +/-3; dorsoventral-4.5 (from dura), with the tooth bar set at -2.3. Hemiparkinsonian rats received a 6-hydroxydopamine (6-OH-DA) injection (9 μg/3 μl dissolved in 0.9% saline containing 0.2 mg/ml ascorbic acid; Sigma) in the right median forebrain bundle at the following stereotaxic coordinates (tooth bar set at -3.3 mm): anteroposterior -3.7 mm; mediolateral -1.6 mm (both from bregma); dorsoventral -8.8 mm from dura. The injection rate was 1 μl/min, and the syringe was kept in place for an additional 5 min before being slowly retracted. Four weeks after the lesion, the rats were tested for rotational behavior in automated rotometer bowls (Panlab) following an injection of apomorphine (0.2 mg/ml; Sigma) and 1 week later with D-amphetamine sulfate (5 mg/kg, intraperitoneally (Sigma); Rotational scores were collected every 2 min for 60 min for D-amphetamine test and 40 min for apomorphine test in a computer-assisted rotometer system (Panlab). Only rats exhibiting 5 or more ipsilateral rotations/min after D-amphetamine injection, and at least 4 contralateral rotations/min in response to apomorphine injection were selected for further unlabeled and MNPs-labeled hVM cell transplantation studies, performed under the same conditions as described above for intact rat brains, but transplanting cells into the right 6-OHDA lesioned striatum . The animals were immunosuppressed with daily intraperitoneal cyclosporin A injection (15 mg/kg; Novartis), starting 2 days before transplantation and throughout the experiment.

For *in vivo* studies, we analyzed the animals at 48 h and from 2 weeks to 5 months following transplantation.

#### Magnetic Resonance Imaging (MRI)

Magnetic resonance imaging (MRI) was performed in the C.A.I. Nuclear Magnetic Resonance and Electron Spin Centre at Complutense University of Madrid. We used the Biospec BMT 47/40 (Bruker, Ettlingen, Germany), which operates at 4.7 Tesla and is equipped with a gradient shield system active of 12 cm. Rats were anesthetized with a mixture of oxygen and isoflurane. Once anesthesized the animals were placed in prone position on a plate of 7 cm in length, head immobilized and connected to a radiofrequency probe to monitor their cardiac and respiratory frequency, and temperature of the animal during image acquisition. First global parameters were determined for centering and optimal collection of images in T2 *. Then, three images of spin echo were run in axial, sagittal, and coronal orientation (TR / TE = 200/10 ms, matrix = 128x128). Transplanted cells labeled with MNPs were visualized performing a gradient-echo sequence using the following parameters: TR = 250 ms, TE = 10 ms, rotation angle = 30 °, thickness of section = 1 mm, number of slices = 8, mean number = 6, FOV = 3x3 cm 2, matrix = 256x192. The reconstructed matrix size was 256 x 256. The acquisition time for these experiments was 4 minutes and 48 seconds. The images were subsequently analyzed with the ImageJ program.

#### Immunohistochemistry

At the end of the experiments, the animals were anesthetized with an overdose of chloral hydrate and intracardially perfused with freshly prepared, buffered 4% paraformaldehyde (in 0.1 M phosphate buffer, pH 7.4). Brains were removed, postfixed for 12 h in the same fixative at 4°C, and dehydrated in 30% sucrose solution at 4°C until sunk. Eleven 30 μm-thick coronal sections were collected using a freezing microtome. Serial sections were used for immunohistochemistry with polyclonal antibodies against human Nestin (1:1000; Abcam), Ki67 (1:500, Neomarkers), Doublecortin (Dcx) (1:1000; Santa Cruz Biotechnology) and monoclonal antibodies against human GFAP (1:1000; Sternberger), human nuclei (hNu) (1:100; Chemicon). Briefly, free-floating sections were incubated overnight at 4°C with the primary antibodies diluted in PBS with 2% nonspecific serum. Sections were rinsed three times in PBS for a total time of 1 h and then incubated for 2 h with the secondary antibodies in PBS (Cy5-conjugated Ab (anti-mouse or anti-rabbit (1:500)), Cy3-conjugated Ab (antimouse or anti-rabbit (1:200), all from Jackson Immunoresearch), and mounted onto polylysine pre-treated glass slides (Menzel-Glaser). The slides were dried and coverslipped with Mowiol.

For staining with DAB, first endogenous tissue peroxidase activity was quenched using a 10% solution of methanol and 3% H_2_O_2_ in PBS for 20 minutes. Subsequently, brain sections were washed with PBS and incubated with blocking solution for 1 h. The sections were then incubated with primary antibody anti OX42 (1:1000, monoclonal, Chemicon) dissolved in a solution of PBS, 0.25% Triton X-100 and 1% horse serum overnight at 4°C. After rinsing, sections were incubated for 2 h with biotinylated secondary antibody (anti-mouse BA2001, dissolved 1:200, Vector) at room temperature. Then sections were incubated with a complex of avidin-biotin-peroxidase (ABC, Vector) and developed with the chromogen 1,3 - diaminobenzidine (DAB, dissolved in 0.05% PBS, 8% NiCl 2 and 0.03% H_2_O_2_ Sigma). Sections were washed in PBS and distilled H_2_O and mounted on slides treated with polylysine, dried at room temperature over night, dehydrated by an increasing gradient of ethanol (50% -90% -100%) and de-lipidatted with xylene. Finally, slides were coverslipped with DPX (BDH).

Analyses and photography of fluorescent or DAB stained samples were carried out in an inverted Zeiss Axiovert 135 (Oberkochen, Germany), LeicaDMIRB microscope equipped with a digital camera Leica DC100 (Nussloch, Germany) or LSM510 laser confocal Microscope coupled to a Axiovert200 (Zeiss) inverted microscope.

## Results

### Internalization of MNPs by VM hNSCs: cell viability, and MNPs size and concentration effects

Different MNPs were compared to select the optimal one to magnetically label VM hNSCs. We tested iron MNPs (Fe_3_O_4_) with different core diameters: 50, 100 and 250 nm, all of them coated with dextran. The influence of different concentrations of MNPs on cell viability was studied at incubation times of 72 h.We also analyzed the effect of the transfection agents, PS and PL, in the case of the lowest concentration of 50 nm MNPs (50 μg/ml), to determine whether the transfection agents could avoid the use of higher doses of NPMs.

**Figure 1:**
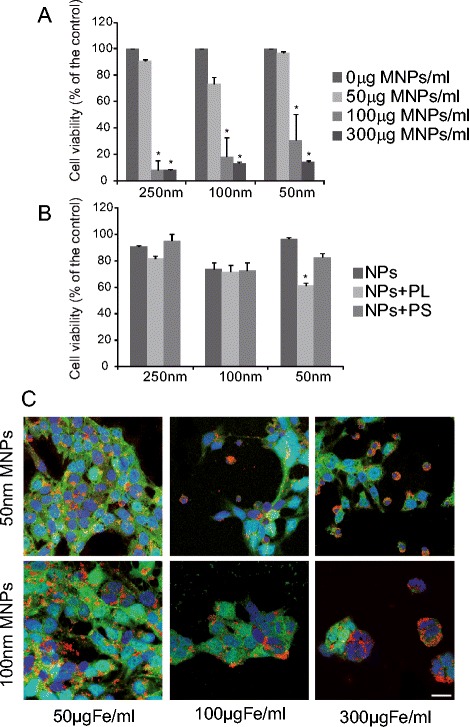
**Cell viability in the presence of MNPs. A**. hVM cells were incubated in the presence of 250, 100 and 50 nm in diameter MNPs for 72 h at increasing concentrations, ranging from 50 to 300 μg/ml. Doses up to 50 μg/ml did not significantly affect cell viability, as evaluated by MTT assay. **B** hVM cell viability (by MTT assay), after 72 h in the presence of 250, 100 and 50 nm MNPs at 50 μg/ml using poly-L-Lysine (PL) and protamine sulfate (PS) as transfection agents. Only PL produced a significant decrease in cell viability. **C** hVM-MNPs-Cy3 treated cells were fixed after 72 h and stained with Phalloidin A488. Nuclei were counterstained with Hoechst. Scale bar 20μm. Data represent mean +/- S.E. (*n* = 4). (*p <0.05, ANOVA, post hoc Tukey’s test; * *versus* hVM cells treated with 0 μg/ml MNPs).

The highest concentrations of nanoparticles tested (100 and 300 μg/ml) produced a significant decrease in cell viability, as evaluated by MTT assay (Figure 1A). Moreover, 50 nm MNPs at 50 μg/ml in the presence of PL produced a significant decrease in cell viability (Figure 1B). In addition to the toxicity observed in the MTT assay, the highest concentrations of particles tested (100 and 300 μg/ml) induced a decrease in the number of cells per field (phalloidin staining), and the presence of numerous apoptotic nuclei stained with Hoechst (Figure 1C).

Thus, labeling of the cells at doses of 50 μg/ml and incubation times of 72 hours did not affect cell survival, evaluated by MTT assay, and resulted in 80 to 90% labeling efficiency (see below). This is valid for all sizes of nanoparticles tested (Figure 1A). These results led us to conclude that labeling hNSCs with MNPs did not significantly affect cell viability, using concentrations of 50 μg/ml for 72 h. Based on these results, we decided to use 50 μg/ml of MNPs for subsequent studies.

### Effects of the incubation time and transfection agents used on the internalization of MNPs by VM hNSCs.

It has been previously reported that cationic transfection agents might improve the efficiency of MNPs intracellular uptake [19,26-29], especially in non-phagocytic cells. Thus, the ability of cationic compounds such as PL and PS might increase the capacity of VM hNSCs to internalize MNPs.

**Figure 2:**
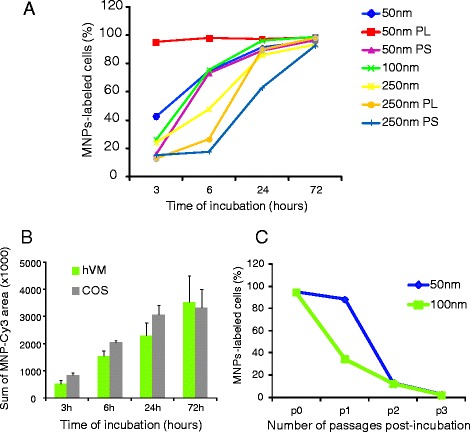
**Incorporation of MNPs by hVM cells and persistence of the MNPs load over time. A**. The incorporation of 50 μg/ml MNPs-Cy3 of 50, 100 and 250 nm in diameter by hVM cells was evaluated as percentage of Cy-3 labeled cells at 3, 6, 24 and 72 h. The effect of poly-L-Lysine (PL) and protamine sulfate (PS) was also evaluated in the uptake of 50 nm and 250 nm MNPs. Incubation times up to 72 h resulted in nearly 100% labeling efficiency even in the absence of PL or PS. **B.** hVM and COS cells were incubated with 100 nm MNP-Cy3 at 50 μg/ml for 3, 6, 24 and 72 hours. The sum of MNP-Cy3 area represents the total area occupied by nanoparticles-Cy3, measured by Image J (NIH) in units of pixels, corrected by the number of cells present in each field. **C.** hVM cells were labeled with 50 and 100 nm MNPs (50 μg/ml) for 72 h and serially subcultured to test the persistence of MNPs labeling after passages. The decreased numbers of MNP-labeled cells observed at progressive subculturing passages indicated that both 50 and 100 nm MNPs are progressively diluted over time.

MNPs uptake was assessed by quantifying the number of cells that displayed fluorescent intracellular labeling of MNPs, as described in the methods section (see also Figure 1C). hVM cells were incubated for different times, ranging from 3 to 72 h, with 50 μg/ml of 50, 100 and 250 nm MNPs, since we had previously demonstrated that this dose did not affect cell viability in most conditions (Figure 1A). The results in Figure 2A show a positive correlation between the uptake of MNPs and the incubation times for all types of MNPs tested. In the case of 50 nm MNPs coated with PL, uptake was maximum at all incubation times studied, indicating that both the size of the particle and the presence of the policationic agent PL significantly influence the time needed to label nearly 100% of the cells. PS had no effect for the 50 nm particles. The largest MNPs tested, 250 nm, were the ones uptaken most slowly by hVM cells, even when PS or PL were added as transfection agents (Figure 2A). To demonstrate an efficient labeling of hVM cells with MNPs, a semi-quantitative assessment (Image J, NIH) of 100 nm MNPs fluorescence signal was performed in hVM cells comparing this signal to a well established SPIO uptake by a common cell line (COS cells) [30] indicating that the uptake of MNPs by hVM cells was similar to that observed in COS cells (Figure 2B).

MNPs with a diameter of 50 and 100 nm were the most appropriate MNPs to label hVM cells at short times. Nevertheless, after a 72 h incubation period, more than 90% of the cells were labeled with MNPs in all conditions tested. The increase in the percentage of labeled cells provided by transfection agents can also be achieved increasing the incubation time with the MNPs. Thus, since these chemical agents induce a decrease in cell viability, especially PL in the case of 50 nm MNPs (Figure 1A), toxicity may be avoided by lengthening the incubation times with the MNPs.

### Persistance of the MNPs load over time

To determine whether the MNPs were retained by hVM cells after passages, cells were incubated with 50 μg/ml of MNPs for 3 days (p0) and then passaged. At each subculturing passage, a fraction of the cells was plated separately and evaluated for the percentage of cells retaining MNP labeling. Dextran staining confirmed a labeling efficiency over 95% at passage 0 (Figure 2C). A progressive drop in the number of dextran-labeled cells with passages was observed, indicating that intracellular MNPs (both 50 and 100 nm sizes), were progressively diluted over time, likely due to cell proliferation. The labeling rate was similar for both nanoparticles 50 and 100 nm after the 2^nd^ and 3^rd^ passages. However, the 50 nm MNPS were retained by the cells much better than the 100nM ones after passage 1 (Figure 2C).

### Effects of MNPs on cell cycle

Analysis of cell replication was determined by fluorescence labeling of the nuclei of the cells in suspension using propidium iodide, and then analyzing the fluorescence properties of each cell in the population by flow cytometry [25]. This study was conducted to determine whether the presence of MNPs was compatible with a normal cell cycle progression in hVM cells. To this end, the cells were exposed to MNPs for 24 and 48 h, and the distribution of cells in the different cell cycle phases was determined.

**Figure 3:**
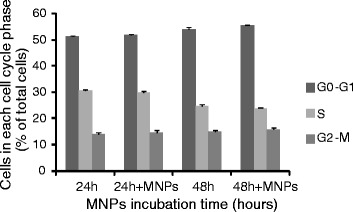
**Effects of MNPs on cell cycle**. hVM cells were either untreated or treated with 50 μg/ml of 100 nm MNPs for 24 and 48 h. Cell cycle analyses were performed by flow cytometry after staining with propidium iodide (PI). The intensity of the PI signal is directly proportional to the DNA content in each phase (G0-G1 phase, S phase, G2-M phase). The percentage of cells in each cell cycle phase showed similar cell cycles profiles for labeled and unlabeled cells.

The percentage of cells in G0-G1, S and G2-M phases was assessed in both control and labeled cells (incubated with 50 μg/ml of 100 nm MNPs). Analysis of the DNA content between samples showed an identical distribution of cells in the different cell cycle phases at both time points (Figure 3). Therefore, MNP labeling of VM hNSCs does not alter cell cycle dynamics. The sub G0-G1 fraction (fragmented DNA) was also quantified to assess cell death by flow cytometry. No cells were detected in subG0 phase in both conditions indicating that the addition of MNPs did not induce cell death, measured as DNA fragmentation (data not shown), consistent with the results of the MTT assay (Figure 1).

### Effects of MNPs on cell stemness and differentiation

**Figure 4:**
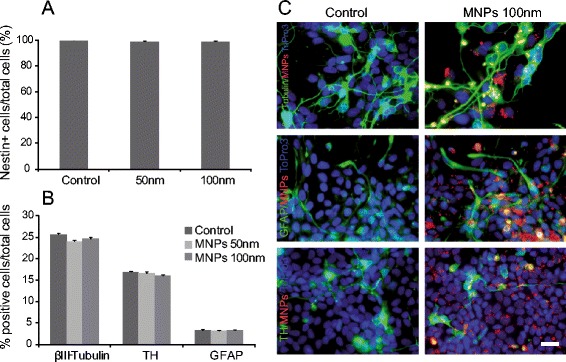
**Effects of MNPs on cell stemness and differentiation. A.** Percentage of nestin positive hVM cells after labeling cells with 50 and 100 nm MNPs at 50 μg/ml for 72 h. **B.** The presence of neurons (β-III-Tubulin+), dopaminergic neurons (TH+) and astrocytes (GFAP+) cells was assessed in hVM cells differentiated for 7 days after labeling them with 50 and 100 nm MNPs at 50 μg/ml for 72 h. **C.** Immunocytochemistry for β-III-Tubulin, TH and GFAP in 7-day differentiated hVM cells either unlabeled (control) or labeled with 100 nm MNPs-Cy3 (in red). Nuclei were counterstained with ToPro3. No significant differences were observed in the relative proportions of the different neural cell types or in their morphology in MNPs-labeled cells with respect to unlabeled cells. Scale bar: 25 μm.

The expression of nestin, a typical marker of neural stem cells, was analyzed in hVM cells labeled with MNPs of different sizes (50 and 100 nm) at 50 μg/ml for 72 h. The percentage of nestin-positive cells in MNPs-labeled hVM cells and in control cells was quantified, and found to be the same in all the studied cultures (Figure 4A). Therefore, MNPs do not induce differentiation themselves.

To assess the differentiation potential of MNPs-labeled hVM cells, after labeling the cultures were subsequently differentiated for 7 days and stained for β-III-tubulin, tyrosine hydroxylase (TH) and glial fibrillary acid protein (GFAP), and counterstained with Hoechst (DNA stain). The percentage of total cells expressing markers characteristic of differentiated VM hNSCs, like β-III-tubulin, GFAP and TH, was compared among samples (Figure 4B, C). No significant differences between control and MNPs-labeled cells were observed, demonstrating that MNPs do not affect the ability of hVM cells to differentiate into glial cells, neurons and, specifically, dopaminergic neurons, obtaining similar results for TH- and β-III-tubulin-positive cells (17% and 25% respectively) to those previously described in [7]. Thus, these results demonstrate that labeling VM hNSCs cells with optimal doses of MNPs does not affect their stemness and differentiation potential.

### Intracellular localization of MNPs in VM hNSCs.

**Figure 5:**
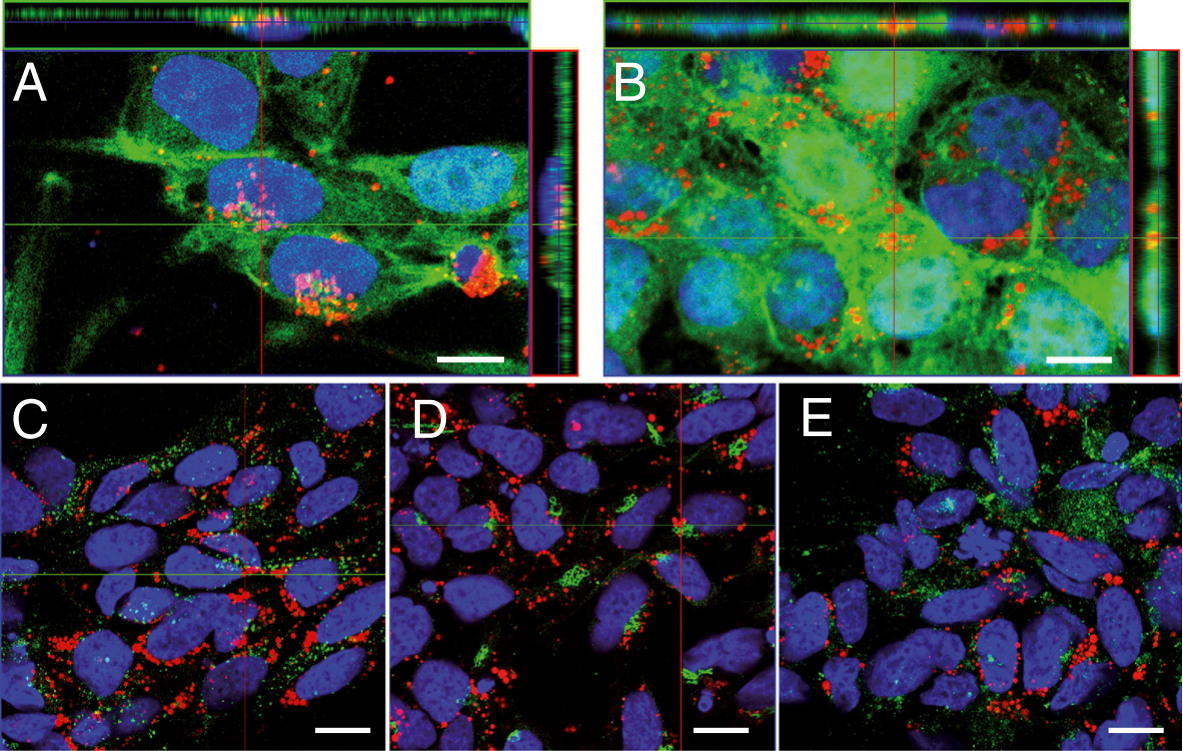
**Intracellular location of MNPs on hVM cells. A-B.** hVM cells were incubated with 50 nm **(A)** and 100 nm MNPs-Cy3 **(B)** at 50 μg/ml for 72 h. After fixation, cells were stained with Phalloidin A488 (green). MNPs in A were detected by staining with an anti-dextran antibody (red). Orthogonal projections are shown in A and B, confirming the colocalization of MNPs and phalloidin. **C-E.** To determine the final intracellular localization of MNPs-Cy3, hVM cells were incubated with 100 nm MNPs-Cy3 at 50 μg/ml for 72 h, antibodies anti-EEA1 specific for early endosomes **(C)**, anti-mannosidase II specific for Golgi apparatus **(D)** and anti-CD63 specific for lysosomes **(E)**, were used. Nuclei were counterstained with ToPro3. Scale bars: 10 μm in A-B and 15 μm in C-E.

To study the subcellular distribution of MNPs uptaken by VM hNSCs, the cells were incubated with 50 and 100 nm MNPs at 50 μg/ml for 72 h, and differentiated afterwards. After that time, proliferation medium was changed to differentiation medium for 4 days. Staining with an anti-dextran antibody or fluorophores directly coupled to MNPs, allowed us to track the intracellular location of the MNPs. To unambiguously determine if the detected NMPs were adhered to the plasma membrane of they were internalized/compartimentalized, series of images were taken along the XZ and XY planes under a confocal microscopy. The orthogonal projections in Figure 5A-B demonstrate that 50 and 100 nm MNPs did not remain superficially attached to cell membranes; on the contrary they were endocytosed by hVM cells.

To determine the final intracellular localization of the MNPs, different fluorescent antibodies, specific for early endosomes (antiEEA1), Golgi apparatus (manoxidase) and lysosomes (CD63) were used. Staining of the endosomes demonstrated that the green signals, labeling endosomes, and the MNPs, labeled in red, are located within the cytosol but do not co-localize (Figure 5C). Golgi apparatus (Figure 5D) and lysosomes (Figure 5E) both stained in green, do not co-localize with MNPs-Cy3 either.

These results indicate that MNPs are internalized by hVM cells labeling the cytosolic compartment, without being sequestered by endosomes, lysosomes or other subcellular organelles, as identified with the markers used in the present assays. In conclusion, the intracellular localization of MNPs in VM hNSCs makes them well suited for MRI analyses.

### MRI analysis of MNP-labeled hVM cells after transplantation

**Figure 6:**
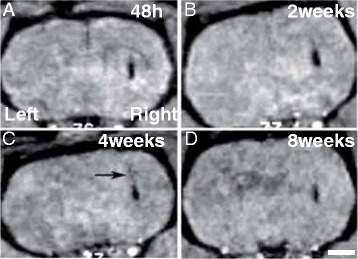
**MR imaging of hVM-MNP labeled cells in rat brain**. Cell suspensions (3x10^5^ cells) were transplanted into the left (hVM cells) and right (hVM-MNP-Cy3 labeled cells) intact striata. MRI was performed 48 h **(A)**, 2 weeks **(B)**, 4 weeks **(C)** and 8 weeks **(D)** after cell transplantation. MNPs can be easily detected by MRI as dark hypointense signals in the area where the MNPs-labeled cells have been injected. No MRI signal was detected when unlabeled hVM cells were transplanted (left striatum). The arrow in **C** shows a small percentage of hVM cells following the injection tract. Scale bar: 2 mm.

Having established an optimized procedure to label hVM cells with MNPs (50 μg/ml of 100 nm MNPs for 72 h) we proceeded to transplant them into right striatum (Figure 6). MRI was performed at different times after cell transplantation (48 h, 2, 4 and 8 weeks). The same number of hVM cells without MNPs were transplanted in the left striatum as an internal imaging control. Strong MRI contrast was observed in the right striatum. No MRI signal was detected when similar numbers of unlabeled cells were injected in the contralateral striatum (Figure 6). The MNPs-labeled hVM cells can be detected from 48 h to 8 weeks after transplantation (Figure 6). 48 h after transplantation, labeled hVM cells are clearly visualized by MRI as a large hypointense signal in the area of the transplant. Although the intensity and size of the magnetic resonance signal decreases slightly during the time periods studied, it is still clearly visible even at 8 weeks after transplantation (Figure 6D). The slight decrease in MRI signal over time might be due to the migration of a small percentage of hVM cells through the corpus callosum that reach this area following the injection tract (arrow in Figure 6C). This observable fact demonstrates that the technique is also valid for tracking small migrating cell populations.

Thus, MRI analysis presents a high spatial resolution and the advantage of visualizing transplanted cells within their anatomical surroundings, which is crucial to check the correct position of the cells after transplantation and the visualization of the cell migration processes.

### Histological analysis of MNPs-hVM cells after transplantation

**Figure 7:**
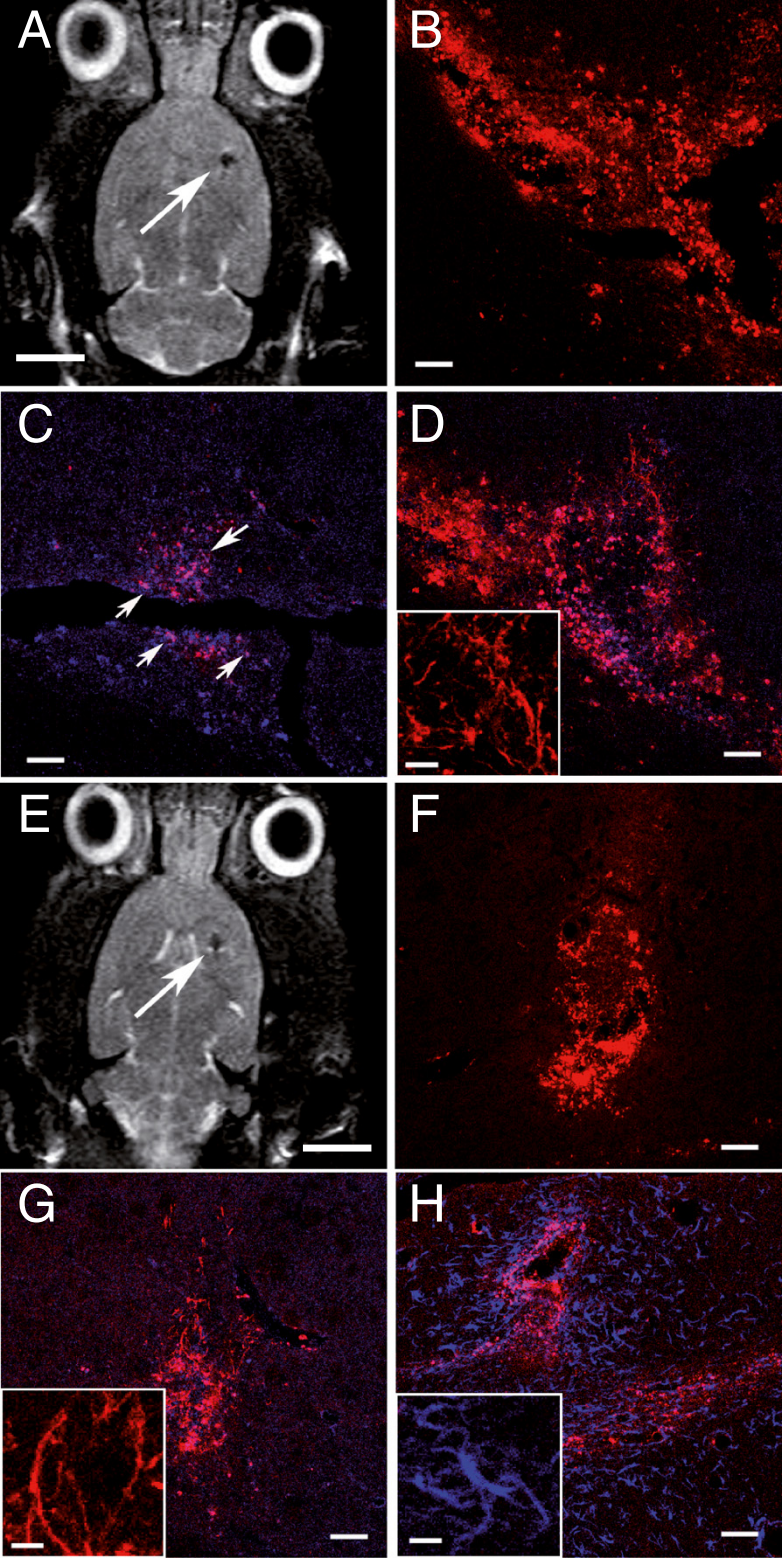
**MR imaging of hVM-MNP labeled cells one week and two months after transplantation into hemiparkinsonian rat brains.** hVM-MNP-Cy3 labeled cells (3x10^5^ cells) were detected by MRI one week (arrow in **A**) and two months (arrow in **E**) after transplantation into the right striatum of 6-OHDA-lesioned rat brains. Analyses by fluorescence microscopy showed that MNPs-Cy3 were clearly visible in red in coronal sections from striatum one week **(B)** and 2 months **(F)** after cell transplantation. Transplants were stained for hNu (blue in **C, D, G**) to detect hVM cells; Ki67 (purple in **C,** arrows point to Ki67 stained cells, showing a purple color, slightly different from MNPs-Cy3 marked in red); human Nestin (red in **D** and **G**, inserts in **D** and **G**) and human GFAP (blue in **H** and insert in **H**). Scale bars: 5 mm in A and E, 50 μm in B-D, F-H; 20 μm in inserts in D, G-H.

The localization of MNPs inside the cells after transplantation was performed using confocal fluorescence microscopy. One week after MNP-hVM cells transplantation in hemiparkinsonian rats, MNPs were identified by MRI scans previous to sacrifice (Figure 7A). Red fluorescent MNPs were evenly distributed throughout the transplant region (Figure 7B). Immunocytofluorescence to detect hVM cells was carried out using an antibody anti-human nucleus (Figure 7C). One week after transplantation, hVM cells present an immature phenotype, as evidenced by the fact that most of these cells are still nestin-positive (Figure 7D). Furthermore, a small percentage of hVM cells are Ki67-positive (Figure 7C), indicating that one week after transplantation some hVM cells are still dividing.

As it has been previously described, two months after transplantation the location of MRI signal is the same to that obtained few hours after transplantation, although there is a slight decrease in signal size and intensity (Figure 6A and D). Analyses by fluorescence microscopy, showed that 2 months after transplantation, the MNPs were aggregated around the limits of the transplant zone, with less intense signal in the graft core (Figure 7F). At this time some hVM cells are still nestin positive (Figure 7G), but some GFAP positive cells are present in the area of the transplant (Figure 7H), indicating some degree of maturation or differentiation in transplanted hVM cells.

### Long term MRI analysis

**Figure 8:**
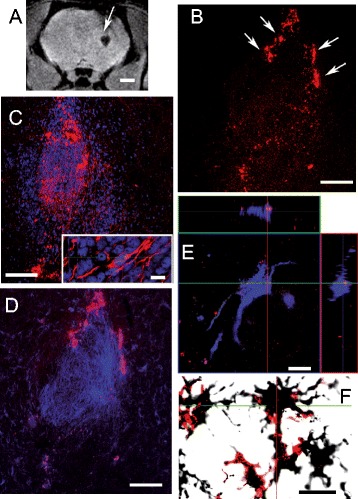
**MR imaging of hVM-MNP labeled cells 5 months after transplantation into hemiparkinsonian rat brains.** hVM-MNP-Cy3 labeled cells (3x10^5^ cells) were detected by MRI 5 months (arrow in **A**) after transplantation into the right striatum of 6OHDA-lesioned rat brains. Analyses by fluorescence microscopy showed that MNPs-Cy3 were clearly visible in red in coronal sections from striatum 5 months after cell transplantation **(B, D)**. Transplants were stained for hNu (blue in **C**) to detect hVM cells; Nestin (red in **C** and insert in **C**); human GFAP (blue in **D** and **E**, orthogonal projections are shown in **E**, confirming the colocalization of GFAP and MNPs-Cy3) and OX42 (stained with DAB) combined with red fluorescence to visualize MNPs-Cy3 **(F)**. Scale bars: 2 mm in A, 100 μm in B-D; 10 μm in insert in C, E-F.

5 months after cell transplantation, a clear hypointense signal at the site of the transplantion is still visible (arrow in Figure 8 A). This signal is quite similar in intensity and size, to that obtained at 2 months after transplantation (Figures 6D and 7E). MNPs maintained their fluorescence five months after transplantation and can be detected in brain sections by fluorescence microscopy (Figure 8B). However, MNPs were found mainly in the boundaries of the transplantation area forming large aggregates outside the cells (arrows in Figure 8B). hVM cells identified by hNu immunoreactivity were predominantly positive for nestin (Figure 8C and insert) and for GFAP (Figure 8D). As expected, the presence of MNPs inside hVM cells was detected, in both nestin (data not shown) and GFAP (Figure 8E). Neurons were not detected in the present experiment. It is important to highlight at this point that human neurons mature very slowly, even when fresh VM tissue is used (over half a year) [31-33]. In fact in other studies using hVM cells we have only observed immature neuronal morphologies [5]. Currently, we are conducting a one-year long experiment to allow the transplant enough time for full maturation.

In the striatum, and especially surrounding the area of the transplant, we can observe the presence of cells of the microglial lineage stained by OX42 (Figure 8F). This microglial reactivity is especially strong in the area surrounding the transplanted MNPs-hVM cells. This OX42 labeling is not observed in brain sections from animals in which unlabeled cells were transplanted (data not shown).The strongest OX42 signal is detected especially in the boundaries of the MNP-hVM cells transplanted area, coinciding with the area where the nanoparticles are more abundantly found. OX42 positive microglial cells co-localized with the signal from MNPs, suggesting that MNPs are removed from the brain parenchyma by microglial cells.

## Discussion

The efficient application of stem cells for the treatment of neurodegenerative diseases requires safe cell tracking to follow stem cell fate over time in the host after transplantation. The present study illustrates the use of commercial nanoparticles to label human neural stem cells without using any transfection agents, for long term tracking by MRI after their transplantation.

In general, most nonphagocytic cells do not take up MNPs efficiently or require cells to be exposed to high amounts of iron in culture [21,22,34-36]. Most magnetic nanoparticles require transfection agents for adequate internalization by stem cells, although it has been reported that Feridex complexed with PL blocked the differentiation of human MSCs into chondrocytes [37]. Only very small superparamagnetic iron oxide particles with a core diameter of 5 nm have been used to label human mesencephalic neural precursor cells [14]. Our data show that nearly 100% of hVM cells were labeled with MNPs of different sizes (50 and 100 nm) in the absence of any transfection agents. These results are similar to those obtained in human NSCs using PS [38] or PL [23,38] as transfecting agents. The present method showed very clean labeling with minimum extracellular iron nanoparticles. These results indicate that with relatively low concentrations of iron (ie, 50 μg of iron per milliliter) in culture media, an almost complete cellular labeling with MNPs can be achieved.

The distribution of cells in the different phases of the cell cycle of MNPs-labeled hVM cells was unaffected, coinciding with findings from other studies, in which MNPs were used to label embryonic stem cells [39], hematopoietic progenitor cells [19] and bone marrow stromal cells [40]. Thus, this procedure that avoids the use of transfecting agents can be considered safe for cell labeling.

We demonstrate the presence of MNPs in the hVM cytosol indicating that MNPs are incorporated by hVM cells via endocytosis. Uptake of 50 nm, 100 nm and 250 nm MNPs at 50 μg/ml occurred in almost 100% of hVM cells, after an incubation period of 72 h without affecting hVM cells survival rate, as evaluated by MTT assay. When using higher concentrations of MNPs (100-300 μg/ml) a significant decrease in cell viability was observed. These results are in agreement with other observations on MNPs-labeling of human NPCs that had no adverse effects on cell survival [23]. However, functional tests in rats transplanted with MNPs-labeled hVM cells must be performed to exclude adverse effect of the MNP labeling on functional outcome of the transplanted rats. Also, before thinking in a future clinical translation, oxidative damage as the one described in mesenchymal stem cells by MNPs [41] should be evaluated, even in the absence of effects of cell proliferation, cell viability and cell death as demonstrated in the present study with hNSCs.

### Loss of MNPs over time

Intracellular MNPs were progressively diluted and almost completely undetectable by immunocytochemistry after 3 subculturing passages. Predicted dilution of MNPs inside hVM cells was most likely due to cell division. Loss of MNPs due to cell proliferation or differentiation has previously been reported for different cell types labeled with different types of MNPs [42-45]. This fact may hinder the reliable long term cell tracking by MRI after cell transplantation [46], in case the cells would have a large replication potential *in vivo*.

### MNPs-labeled hVM cells maintain their stemness and differentiation potential

The feasibility of cell-based therapies depends upon the ability of transplanted cells to maintain their proposed therapeutic functions *in vivo*. Previous studies have revealed that hVM cells differentiate into a high percentage of dopaminergic neurons [8,9]; this characteristic makes them particularly attractive for cell replacement therapy in Parkinson’s disease. It has been also demonstrated that most stem cells labeled with MNPs retain their regenerative and therapeutic potential in vivo [38,47]. In order to assess that the optimized labeling protocols did not affect the stemness or differentiation potential of hVM cells, we analyzed the presence of nestin, a typical marker of neural stem cells, in cells treated with MNPs compared with control unlabeled cells. As described in the Results section, the labeling of hVM cells with MNPs did not affect lineage commitment during the differentiation period of hVM cells. We have analyzed the percentage of TH^±^ neurons generated after 7 days of differentiation. Labeling hVM cells with MNPs did not affect the percentage of TH^+^ cells obtained during the differentiation process. We did not observe any significant changes in the percentage of nestin, GFAP or β-III-tubulin hVM cells compared to MNPs-hVM cells, after 7 days of differentiation. These results confirm previous data reported in human [14,23,48] and mouse [12] neural precursor cells labeled with ferumoxides. However, it has been observed that MNPs could cause morphological changes and alterations in the differentiation pattern of human adult mesenchymal stem cells [37,49] but not in mouse mesenchymal stem cells [50]. In the present study with hNSCs, we did not observe any morphological alteration of the labeled and differentiated cells in vitro. On the contrary to these findings, others have demonstrated an increase in GFAP expression and changes in cell morphology when treating hNPCs with Au- and Ag-nanoparticles [51].

Using confocal microscopy, the nanoparticles did not co-localize with any of the markers of the subcellular organelles analyzed (endosomes, lysosomes, endoplasmic reticulum or Golgi apparatus). This suggests that these nanoparticles can be freely distributed in the cytoplasm and eventually enter the normal iron metabolic pathways [26]. However, further electron microscopy analysis must be carried out to unequivocally determine the exact subcellular localization of the nanoparticles.

### MRI and hVM cells

Previous studies have shown that magnetically labeled cells maintained their contrast for 6 weeks [44], 1 month [47] and even 3 months after transplantation [14], suggesting that labeling cells with MNPs can be a suitable method for cell tracking by MRI over time. However, long term analysis after cell transplantation might be deceiving, especially if transplanted cells continue dividing in the host brain [52]. As shown here, MNPs-labeled cells show a gradual reduction in intracellular iron particles after 2 or 3 passages in cell cultures. Therefore, dilution but also the loss of MNPs from transplanted cells to the host brain parenchyma might occur during cell division. MNPs released to the host parenchyma during cell division, exocytosis processes or even after cell death, can contribute to the MR signal.

In short term MRI analyses (1 week and 2 month after cell transplantation) we observed an excellent agreement between the areas of MR contrast enhancement and histological staining for nanoparticles. Therefore, we can conclude that this technique could also be valid to assess the precise position of the grafted cells shortly after transplantation.

However, long term MRI analyses are needed to perform because transplanted cells are expected to slowly mature into the correct phenotypes and participate to some extent in tissue reconstruction (over half a year). Our results showed that there were still some nestin positive hVM cells at 5 months post-transplantation. Although at that time we found numerous GFAP positive cells, grafted cells mainly express progenitor markers at short times after transplantation [45]. The ability of human embryonic stem cell neurons to provide extensive reinnervation of the host striatum and begin to restore DAergic neurotransmission have been described at least 6 months post-transplantation [53]. This is in line with previous clinical observations using human fetal VM grafted to patients, where progressive recovery of DA neurotransmission starts at 6 months, reflecting the gradual maturation of the transplanted cells [54]. Similar results have been recently described when using human pluripotent stem cells (hPSCs). Functional maturation of hPSC-derived neurons requires an extended timeline of up to seven months, mimicking endogenous human neural development [55].

Although some studies found no co-localization of mouse macrophages and iron-containing areas of brain tissue [56] this event has been described previously in a spinal cord injury model transplanted with Endorem-labeled mesenchymal stem cells [57]. In our case, long term MRI analyses followed by histological analyses (up to 5 months after cell transplantation) showed that MNPs can be found in the brain parenchyma co-localizing with areas rich in markers of reactive microglia, like OX42. This fact is a cause of concern because of the possible false positive interpretation of the MRI signal, which may be produced by macrophages that have engulfed some non-viable labeled NSCs or freely-dispersed iron nanoparticles in the brain tissue [58].

## Conclusion

NSCs are one of the most attractive cells sources for stem cell therapies of neurodegenerative diseases. For therapeutic application, transplanted cells need to be tracked both spatially and temporally in the living brain, in order to assess their migration and survival in the host tissue. Therefore, for the application of these techniques *in vivo*, additional live imaging tools such as MRI are necessary. The procedures described here are suitable for studying the *in vivo* localization and migration of grafted human neural stem cells in longitudinal studies. We have shown that labeling of hVM cells with MNPs does not affect their overall biology, survival rate and *in vitro* differentiation. These findings support the use of commercial MNPs to track cells for short- and mid-term periods after transplantation for studies of brain cell replacement therapy. Nevertheless, long-term MR images should be interpreted with caution due to the possibility that some MNPs may be expelled from the transplanted cells and internalized by host microglial cells.
